# The Characterization of SaPIN2b, a Plant Trichome-Localized Proteinase Inhibitor from *Solanum americanum*

**DOI:** 10.3390/ijms131115162

**Published:** 2012-11-16

**Authors:** Ming Luo, Ling-Wen Ding, Zhi-Juan Ge, Zhen-Yu Wang, Bo-Lun Hu, Xiao-Bei Yang, Qiao-Yang Sun, Zeng-Fu Xu

**Affiliations:** 1 State Key Laboratory of Biocontrol and Key Laboratory of Gene Engineering of the Ministry of Education, School of Life Sciences, Sun Yat-sen University, Guangzhou 510275, China; E-Mails: luomingjs@yahoo.com.cn (M.L.); zipy@163.com (L.-W.D.); iawsfi@yahoo.com (Z.-J.G.); wangzyjia@126.com (Z.-Y.W.); tyd0898@sina.com.cn (B.-L.H.); belinda0213@163.com (X.-B.Y.); qiaoyang1983@163.com (Q.-Y.S.); 2 Key Laboratory of Plant Resources Conservation and Sustainable Utilization, South China Botanical Garden, Chinese Academy of Sciences, Guangzhou 510650, China; 3 Key Laboratory of Tropical Plant Resource and Sustainable Use, Xishuangbanna Tropical Botanical Garden, Chinese Academy of Sciences, Menglun, Yunnan 666303, China

**Keywords:** PIN2, proteinase inhibitor, *Solanum americanum*, insect resistance, trichome

## Abstract

Proteinase inhibitors play an important role in plant resistance of insects and pathogens. In this study, we characterized the serine proteinase inhibitor SaPIN2b, which is constitutively expressed in *Solanum americanum* trichomes and contains two conserved motifs of the proteinase inhibitor II (PIN2) family. The recombinant SaPIN2b (rSaPIN2b), which was expressed in *Escherichia coli*, was demonstrated to be a potent proteinase inhibitor against a panel of serine proteinases, including subtilisin A, chymotrypsin and trypsin. Moreover, rSaPIN2b also effectively inhibited the proteinase activities of midgut trypsin-like proteinases that were extracted from the devastating pest *Helicoverpa armigera*. Furthermore, the overexpression of SaPIN2b in transgenic tobacco plants resulted in enhanced resistance against *H. armigera*. Taken together, our results demonstrated that SaPIN2b is a potent serine proteinase inhibitor that may act as a protective protein in plant defense against insect attacks.

## 1. Introduction

In nature, a large number of plants respond to wounding or insect attack through the production of defense proteins, such as proteinase inhibitors (PIs), lectins or chitinases [1]. PIs are widely distributed throughout the plant kingdom [2–7] and have been extensively investigated [8–10]. PIs are grouped into four families according to the four types of proteinases that they inhibit, which include serine proteinases, cysteine proteinases, aspartate proteinases and metalloproteinases; these classifications are based on the active amino acid that is located in the “reaction center” of these proteinases [11]. The most extensively studied proteinase inhibitors in plants are serine and cysteine proteinase inhibitors. Based on the amino acid sequences of plant serine proteinase inhibitors, these inhibitors have been divided into eight families [12]. The soybean trypsin inhibitor (SKTI), proteinase inhibitor 1 (PIN1) and proteinase inhibitor 2 (PIN2) families have been well studied. PIN2 family contains four groups: the Kunitz, Bowman Birk, Potato I and Squash families. PIN2 enzymes demonstrate wound-inducible expression patterns in leaves and constitutive expression in flowers.

PIs play a potent defensive role against predators and pathogens [13,14]. PIs have been used as protective genes for the development of insect-resistant transgenic plants [15–17], which were obtained from crop plants, such as rice, barley, soybean cowpea, sweet potato and maize [18–20]. However, a large number of insect pests have evolved adaptations to their host plant PIs, and transgenic crop plants did not evince enhanced resistance to these pests [21,22]. Evidence has shown that PIs from nonhost plants can reliably inhibit the midgut proteinases of crop pests [23]. Therefore, it is important to identify new PIs that have evolved separately from the insect pests of crop plants.

We have previously isolated two PIN2 cDNAs (*SaPIN2a* and *SaPIN2b*) from the nightshade *Solanum americanum*, a weed belonging to the Solanaceae family, which are differentially expressed in plants [24,25]. SaPIN2a is abundantly expressed in phloem [24], whereas *SaPIN2b* is constitutively expressed in glandular trichomes [25]; and the overexpression of *SaPIN2a* and *SaPIN2b* in transgenic plants resulted in a significant increase in glandular trichome density and the promotion of branching [25,26]. These observations prompted us to further investigate the biochemical properties and biological functions of SaPIN2a and SaPIN2b. In contrast to SaPIN2a, which has been previously purified and characterized [27], the purification of native SaPIN2b is difficult because of the limited amount of plant trichomes. Therefore, in this study, we used a glutathione S-transferase (GST) fusion tag in an *E. coli* BL21 expression system to produce recombinant SaPIN2b and assessed the inhibitory activity of purified rSaPIN2b against a panel of serine and cysteine proteinases. Our results indicated that SaPIN2b could inhibit subtilisin A, chymotrypsin and trypsin through a noncompetitive mechanism. However, no inhibitory activity was observed against either of the cysteine proteinases cathepsin D or papain. Moreover, rSaPIN2b effectively inhibited the midgut trypsin-like proteinase activity of *Helicoverpa armigera*, a devastating pest that causes severe damage to plant crops worldwide and uses serine proteinases as major digestive enzymes [28]. To further explore the potential role of SaPIN2b in plant defense against insects, the larvae of *H. armigera* were applied to the leaves of SaPIN2b-overexpressing transgenic tobacco plants. Our results indicated that SaPIN2b was indeed a potent inhibitor of serine-type proteinases that could significantly enhance insect resistance in transgenic plants.

## 2. Results and Discussion

### 2.1. The Expression and Purification of Recombinant SaPIN2b

**Figure 1:**
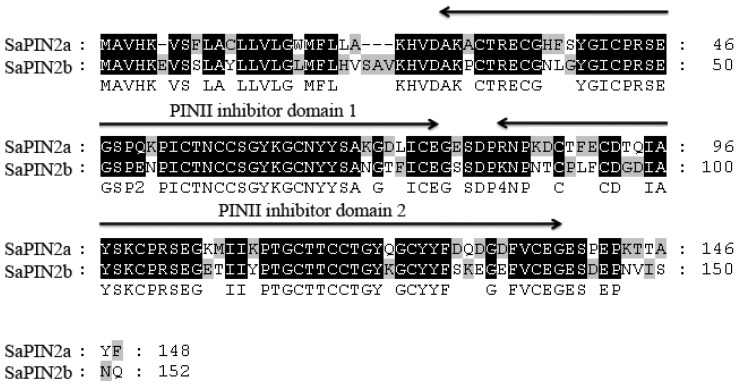
A comparison of the amino acid sequences of SaPIN2a and SaPIN2b from *Solanum americanum*. The sequence alignment was performed using ClustalX software [29], and the sequences were subsequently adjusted using GeneDoc software. The shaded blocks indicate consensus sequence motifs. The horizontal arrows indicate PINII inhibitor domains, which were explored using the Pfam databases [30].

**Figure 2:**
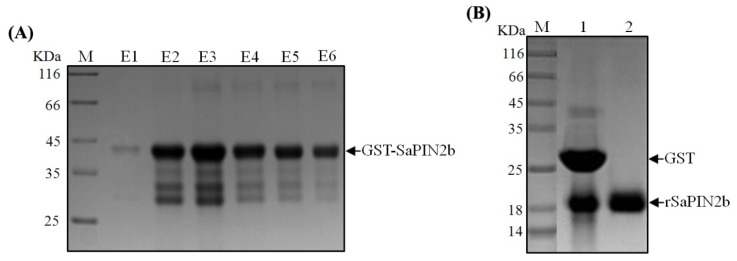
The expression and purification of recombinant SaPIN2b protein. (**A**) An SDS-PAGE gel of the GST-SaPIN2b fusion protein that was purified from the GSTrap^™^ column. M: Protein Marker (Fermentas, SM0431). Lanes E1-E6: The eluate was collected in tubes 1–6 (0.5 mL/tube), and 10 μL of each eluted fraction was loaded onto the gel. The arrow indicates the size of the GST-SaPIN2b protein; (**B**) The SDS-PAGE gel of rSaPIN2b. M: Protein Marker (Fermentas, SM0431). Lane 1: GST-SaPIN2b that had been digested with 0.75 U/mg thrombin for three hr. Lane 2: rSaPIN2b protein was purified on an agarose-trypsin affinity column. The sizes of GST and rSaPIN2b are indicated with arrows.

The full-length cDNAs that encode the proteinase inhibitors SaPIN2a and SaPIN2b have been previously isolated from *S. americanum*[24,25]. A comparison of the amino acid sequences of SaPIN2a (GenBank accession number AF174381) and SaPIN2b (GenBank accession number AF209709) was performed using ClustalX software (Figure 1). These two proteins share 73.6% amino acid sequence identity and contain two conserved PINII inhibitor domains (Figure 1). We have previously demonstrated that SaPIN2a exhibits proteinase inhibitor activity in either native or recombinant form [25]. Because of difficulties in purifying native SaPIN2b, the proteinase inhibitor activity of SaPIN2b was examined using a recombinant protein containing mature SaPIN2b that had been fused in-frame with an N-terminal GST tag. The GST-SaPIN2b fusion protein was expressed in *E. coli* BL21. A time course of protein expression revealed that the maximum expression of recombinant GST-SaPIN2b was achieved at three hours after its induction at 28 °C with 0.4 mM IPTG. After this induction, rSaPIN2b was purified using two-step chromatography. First, the GST-SaPIN2b fusion was purified using a GSTrap^™^ column (Figure 2A); subsequently, the *N*-terminal GST tag was removed through thrombin digestion (Figure 2B). The rSaPIN2b protein was then further purified using an agarose-trypsin affinity column. After the purified rSaPIN2b had been eluted from the trypsin affinity column, it appeared as a single, dark band in SDS-PAGE analyses (Figure 2B). This result indicated that rSaPIN2b was successfully expressed and purified.

### 2.2. Recombinant SaPIN2b as a Potent Serine Proteinase Inhibitor

**Figure 3:**
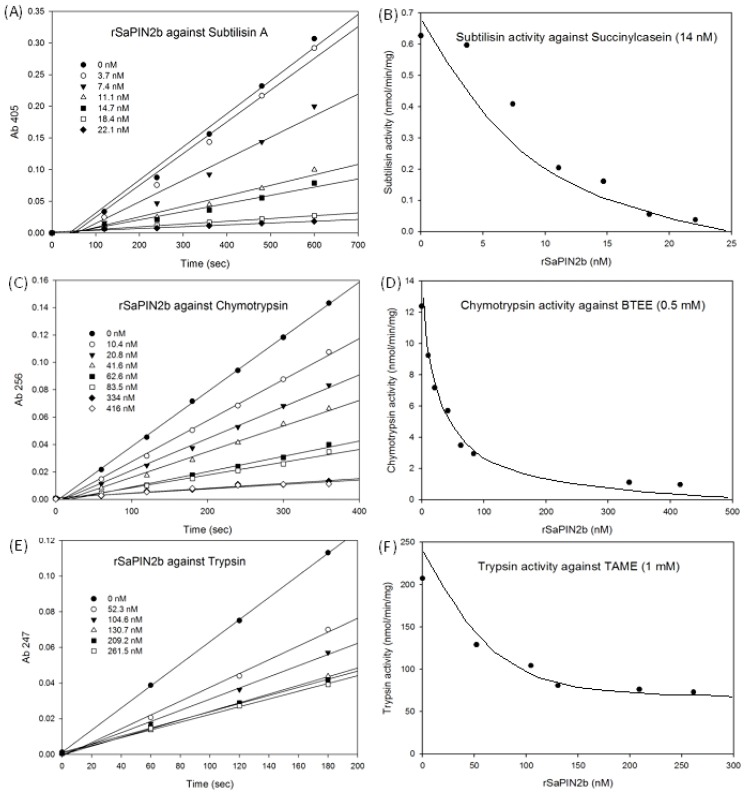
Analysis of the inhibition of various serine proteinases by rSaPIN2b. The time course of the inhibitory effect of rSaPIN2b against subtilisin (**A**), chymotrypsin (**C**) and trypsin (**E**). The remaining proteinase activity at the final reaction time point with different concentration of rSaPIN2b was shown in (**B**) for subtilisin, (**E**) for chymotrypsin, and (**F**) for trypsin, respectively. The substrates that were used in the assays were 14 nM succinylcasein (**A**, **B**), 0.5 mM BTEE (**C**, **D**), and 1.0 mM TAME (**E**, **F**).

To further characterize the inhibitory activity of SaPIN2b, its effects on a panel of different proteinases were examined. As shown in Table 1 and Figure 3, rSaPIN2b demonstrated strong inhibitor activities to various serine proteinases and appeared to be a potent inhibitor of subtilisin A (IC_50_ = 7.3 nM), chymotrypsin (IC_50_ = 34.9 nM) and trypsin (IC_50_ = 126.7 nM). Consistent with the previously observed results for SaPIN2a, no inhibition was observed against the cysteine proteinases papain and cathepsin D (data not shown) [25]. Therefore, this result demonstrated that SaPIN2b is a potent serine proteinases inhibitor. Previously, the fully functional potato PIN2 had only been expressed in yeast because this protein was incorrectly folded when expressed in *E. coli*[31]. Similarly, the mustard trypsin inhibitor with four disulfide bonds has been successfully expressed in yeast but not in *E. coli*[32]. However, in this study, the recombinant SaPIN2b protein that demonstrated inhibitory activity was successfully expressed and purified in an *E. coli* system. Taken together, these data suggest that the expression of active PIN2 may be accomplished through both prokaryotic and eukaryotic expression systems.

### 2.3. Enzymatic Assays to Test rSaPIN2b Against Digestive Proteinases That Have Been Obtained from the Midgut of *Helicoverpa armigera* Larvae

**Figure 4:**
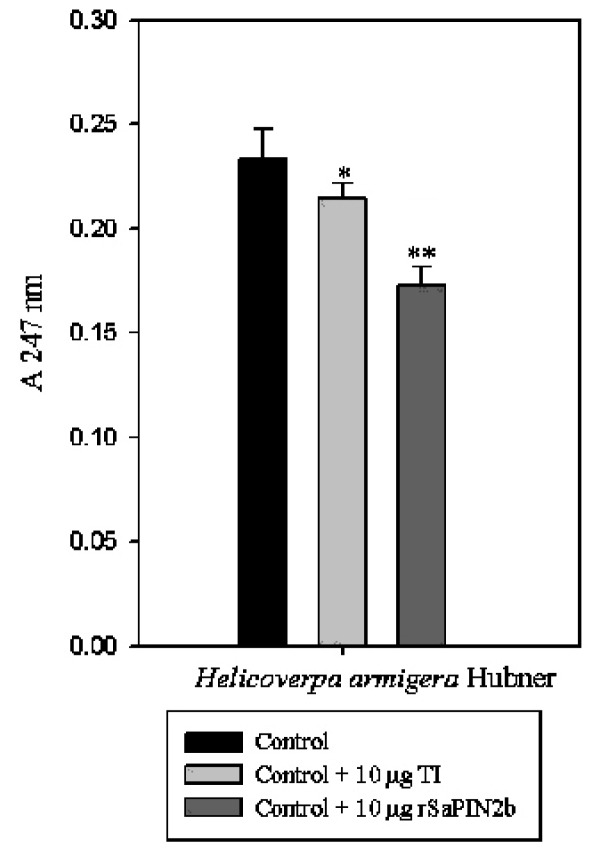
Inhibitory activities of rSaPIN2b against trypsin-like proteinases from the midgut of *Helicoverpa armigera*. A total of 200 μg of protein was extracted from the midgut of *H. armigera*, and 10 μg of either rSaPIN2b or the SBTI) were incubated for 3 min at 37 °C. Proteolysis was determined through the addition of the TAME substrate.

The midgut proteinases of *H. armigera* have been primarily identified as serine proteinases, and trypsin-like proteinase activity has also been observed in the midgut of *H. armigera*[26,28]. Our previous studies have revealed that both native and recombinant SaPIN2a proteins exhibit inhibitory effects on trypsin-like activity in the midgut of *H. armigera*[27]. To explore the potential of SaPIN2b for developing insect-resistant transgenic plants, the inhibitory activity of SaPIN2b against the trypsin-like proteinase activity in the midgut of *H. armigera* was analysed using TAME, a trypsin substrate. The inhibitory activity of rSaPIN2b against trypsin-like proteinases from the *H. armigera* midgut was 20% greater than the activity of the well-known soybean trypsin inhibitor SBTI (Figure 4). Our observation implies that rSaPIN2b is a potent inhibitor of insect midgut and suggests that SaPIN2b could be a potential inhibitor for developing insect-resistant transgenic plants.

### 2.4. The Overexpression of SaPIN2b in Transgenic Tobacco Plants Enhanced the Resistance of These Plants to *Helicoverpa armigera*

**Figure 5:**
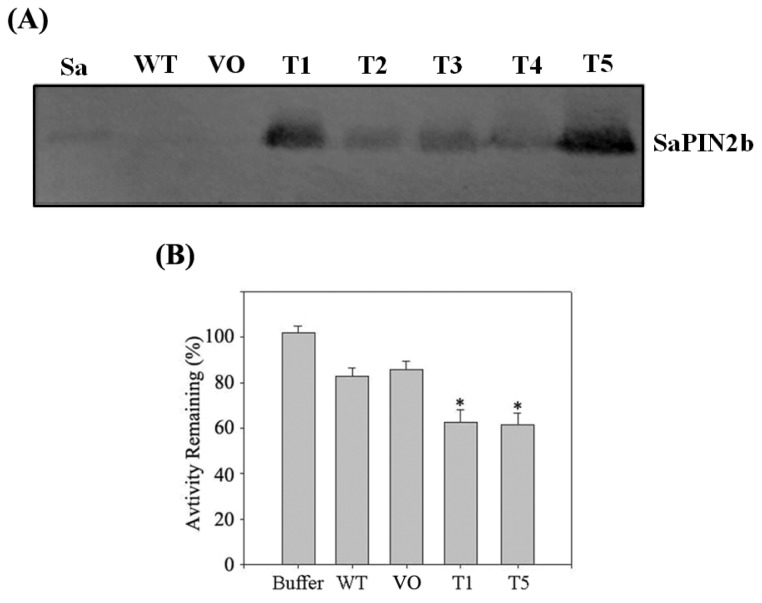
The overexpression of SaPIN2b in transgenic tobacco plants. (**A**) The Western blot analysis of SaPIN2b-overexpressing tobacco plants. Sa, total proteins were extracted from the stems of *S. americanum* (positive control); WT, wild-type tobacco plants (negative control); OV, vector-only transgenic tobacco plants (negative control); and T1-T5, SaPIN2b-overexpressing plants. (**B**) The inhibition of trypsin-like proteinases from the midgut of *H. armigera*. Total soluble proteins (TSP) extracted from tobacco leaves were used in this assay. Buffer, extraction buffer for TSP; WT, wide-type tobacco plants; VO, vector-only transgenic tobacco plants; T1 and T5, SaPIN2b-overexpressing plants. * indicates that *p* < 0.05. (The data were analyzed with a *t*-test).

It was demonstrated that transgenic plants expressing potato or tomato PIN2 show an enhanced resistance to herbivorous insects [15,33]. In our laboratory, we have previously shown that the overexpression of SaPIN2a in transgenic tobacco plants enhanced the resistance of these plants to two devastating pests, *H. armigera* and *S. litura*[26]. In this study*,* we generated tobacco transgenic plants that overexpressed the SaPIN2b gene under the control of the strong constitutive CaMV35S promoter. Independent T_3_ generation transgenic lines were screened by PCR. Western blotting using SaPIN2b-specific antibodies was performed to detect the accumulation of SaPIN2b proteins in the leaves of transgenic tobacco plants. As shown in Figure 5A, a clear band corresponding to SaPIN2b protein was detected in *S. americanum* and in the transgenic tobacco plants that overexpressed SaPIN2b, whereas this band were absent in WT and vector-only (VO) control tobacco plants. This result indicates that the SaPIN2b proteins were successfully overexpressed in transgenic tobacco plants. To further examine the inhibitor function of SaPIN2b in transgenic plants, crude proteins were extracted from the leaves of SaPIN2b transgenic tobacco plants, and consistent with the results obtained with rSaPIN2b, the T1 and T5 homozygous transgenic plants demonstrated higher inhibitory activity than control plants against trypsin-like proteinases from the midgut of *H. armigera* (Figure 5B).

**Figure 6:**
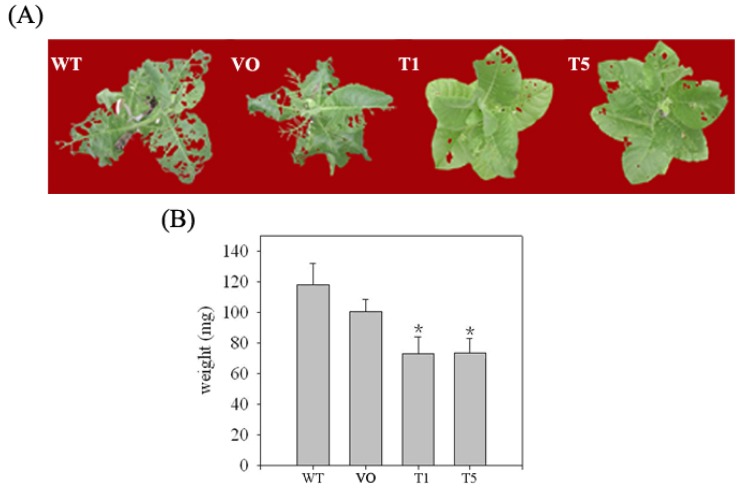
Insect bioassays of SaPIN2b-overexpressing transgenic plants. (**A**) Each tobacco plant was infected with 10 early second-instar *H. armigera* larvae for seven days. WT, wild-type plants; VO, vector-only transgenic plants; T1 and T5, SaPIN2b-overexpressing plants. (**B**) Weight of *H. armigera* fed on different tobacco lines. * indicates that *p* < 0.05. (The data were analyzed using the *t*-test).

Subsequently, the screened transgenic lines were used for insect feeding experiments. As shown in Table 2, significantly higher mortality was observed for *H. armigera* that fed on SaPIN2b-overexpressing plants than for *H. armigera* that fed on WT or vector-only transgenic plants, and more severe damage was observed on the leaves of the WT and vector-only transgenic tobacco plants than on the SaPIN2b overexpressing transgenic plants (Figure 6A). The average weight of the insects that fed on the WT and vector-only transgenic plants was significantly higher than the average weight of the insects that fed on the SaPIN2b-overexpressing transgenic plants (Figure 6B). Furthermore, the pupation rate of the *H. armigera* larvae that fed on SaPIN2b-overexpressing transgenic leaves was 38.3–41.1%, whereas the pupation rate of the *H. armigera* larvae that fed on the two different types of control plants was 78.6–81.5% (Table 2). These results suggest that SaPIN2b overexpression in transgenic tobacco plants enhanced the resistance of these plants to *H. armigera*.

### 2.5. Discussion

Many plants respond to insect attack by producing defense proteins, like lectins, chitinases, or proteinase inhibitors, which have been extensively investigated in the past several decades [34,35]. At the same time, the insect herbivores also develop different approaches to handle these plant defense proteins and eventually adapt to their host plants [36]. The “arm race” between the insect proteinases and host proteinase inhibitors provides interesting examples of the coevolution between insects and their host plants [37].

PIN2 type proteinase inhibitors have been well characterized as wound-induced defense proteins, since their first discovery in tomato and potato [38]. Subsequent studies show that the Jasmonate signaling pathway mediated the wound-induced expression of PIN2s [39,40]. Interestingly, our previous study showed that SaPIN2b was expressed constitutively in glandular trichomes, and overexpression of SaPIN2b in tobacco resulted in a significant increase in glandular trichome density and promotion of trichome branching [25]. Glandular trichomes provide the first line of defense against insects by entrapping or impeding small herbivores [41], and also function as an early detection system for insect herbivores [42]. In this study, we showed that overexpression of SaPIN2b in transgenic tobacco enhanced plant resistance to *H. armigera*, which may result from the combined effects of the increased inhibitory activity against the proteinases from insect midguts and the much more glandular trichomes on leaves of transgenic plants.

## 3. Experimental Methods

### 3.1. RNA Isolation and RT-PCR

An RNeasy Plant Mini Kit (Qiagen) was used in accordance with the manufacturer’s protocol to isolate total RNA from *Solanum americanum* flowers. Based on GenBank sequence number AF209709, the full-length cDNA encoding SaPIN2b, including the 5′ and 3′UTR regions, was amplified from first-strand cDNA, using the primer sequences for 2b5UTRpan (5′-TTAATCACGATCGAGAAAGAATA-3′) and 2b3UTRpan (5′-GTGATTATCATATATTGCCGTAC-3′). The PCR product was ligated into the pGEM-T Easy Vector (Promega) to produce vector pT2b; successful ligation was verified through sequencing.

### 3.2. The Expression and Purification of Recombinant SaPIN2b

To generate GST-tagged versions of recombinant SaPIN2b, a pair of primers, ZF303 (5′-GCGAATTCGTAAAACATGTTGAT-3′) and ZF304 (5′-GGCTCGAGTAAA-AACTACAACGC-3′), that contained *Eco*RI and *Xho*I restriction sites were designed to amplify SaPIN2b from pT2b. The amplified fragment was digested with *Eco*RI and *Xho*I restriction enzymes and subcloned into the pGEX-4T-1 expression vector (Amersham). After the successful construction of the desired plasmid was verified through sequencing, the plasmid was transformed into the *E. coli* protein expression strain BL21(DE3).

*E. coli* BL21 was transformed with the expression vector and grown in 2 × YT supplemented with 100 μg/mL of ampicillin at 37 °C until the OD_600_ reached 0.5; the transformed bacteria were then induced through the addition of IPTG to a final concentration of 0.4 mM. After incubation for 3 h at 28 °C, the cells were harvested using centrifugation, and the pellet was suspended in PBS (140 mM NaCl, 2.7 mM KCl, 10 mM Na_2_HPO_4_, 1.8 mM KH_2_PO_4_; pH 7.4). After cell disintegration, the cell suspension was centrifuged at 10,000 × *g* for 30 min at 4 °C. To purify the GST-SaPIN2b fusion protein, the soluble cell extract was applied to a GSTrap^™^ column (Amersham) in accordance with the manufacturer’s instructions.

For the further removal of the *N*-terminal GST tag, the purified fusion protein was treated with thrombin (Novagen) at 22 °C for 3 h. The rSaPIN2b was purified using the method developed by Murray [43], with minor modifications. The digested mixtures were applied to an agarose-trypsin affinity column (Immobilized TLCK-trypsin, Pierce). The column was washed with 10 bed volumes of equilibration buffer (150 mM KCl/10 mM Tris-HCl; pH 8.0), and the bound proteins were eluted with elution buffer containing 10 mM HCl, 30 mM CaCl_2_ and 200 mM NaCl (pH 2.0); the eluate was immediately neutralized with 1 M Tris base (pH 9.0).

### 3.3. The Production of SaPIN2b Polyclonal Antibody

To generate the anti-SaPIN2b antibody, recombinant SaPIN2b was excised from an SDS-PAGE gel and used as an antigen to inject rabbits for antibody production. An ImmunoPure Immobilized Protein A (Pierce) affinity column was used in accordance with the manufacturer’s instructions to further purify the antibodies that were generated.

### 3.4. The Determination of the Protein Concentration

In accordance with previously described procedures [44], protein concentrations were determined using a Micro BCA protein assay kit (Pierce) or the Bradford method; bovine serum albumin was used as a standard.

### 3.5. Proteinase Inhibition Assays and Kinetic Analysis

Inhibitory activity of rSaPIN2b against subtilisin A was determined with substrate succinylcasein (Calbiochem Cat. No. 573464) following the previously described method [45]. Inhibitory activity of rSaPIN2b against chymotrypsin and trypsin was determined with substrate *N*-benzoyl-l-tyrosine ethyl ester (BTEE, Sigma, St. Louis, MO, USA) and Nα-p-tosyl-l-arginine methyl ester hydrochloride (TAME, Sigma, St. Louis, MO, USA) respectively, according to the method described by Kollipara *et al.*[46]. Inhibitory activity of rSaPIN2b against Cathepsin D and papain was assayed by the previously described method [47,48].

### 3.6. The Extraction and Activity Assay of Proteinases from Insect Midguts

The crude midgut proteins were prepared from fifth-instar *H. armigera* larvae in accordance with previously described methods [27]. The inhibitory activities of rSaPIN2b toward trypsin-like proteinases of the insect midguts were assessed by the method of Wang *et al.*[27], and the soybean trypsin inhibitor (SBTI, Calbiochem, La Jolla, CA, USA) was used as a control. Similarly, the inhibition of leaf extracts from SaPIN2b transgenic tobacco was also assessed using the method described above.

### 3.7. The Generation of SaPIN2b-Overexpressing Transgenic Plants

To generate a plant overexpression vector for SaPIN2b, the plasmid pT2b was digested with *Kpn*I and *Bam*HI; subsequently, the similarly digested SaPIN2b cDNA was inserted into the *Kpn*I and *Bam*HI restriction sites of the pHANNIBAL vector[49], resulting in an intermediate vector, pH2b, that was then digested further with *Not*I. This fragment, which was composed of the 35S promoter, SaPIN2b cDNA and the Ocs terminator region, was subsequently inserted into the PART27 binary vector [50], which had previously been digested with *Not*I. After sequencing, the SaPIN2b expression vector was transformed into *Agrobacterium tumefaciens* strains LBA4404 [51]. SaPIN2b-overexpressing transgenic tobacco plants were generated using the *Agrobacterium tumefaciens*-mediated method that was described by Horsh *et al.*[52]. The transgenic plants were germinated and selected on Murashige and Skoog agar medium containing 100 μg/mL of kanamycin and subsequently transferred to soil and grown in a greenhouse.

### 3.8. Western Blot Analysis

Western blot analysis was performed according to the procedures of Xu *et al.*[53]. Anti-SaPIN2b was used as a primary antibody, and the resulting signals were detected using the Goat Anti-Rabbit Immuno-Blot Assay Kit (Bio-Rad).

### 3.9. Insect Feeding Trials

T_3_ homozygous SaPIN2b transgenic plants (lines T1 and T5) were used in the insect feeding trials, and the WT and vector-only transgenic plants were used as controls. All of the plants were grown simultaneously under greenhouse conditions. The *H. armigera* larvae were obtained from Jiyuan Baiyun Industry Company, Ltd. (Henan, China). The insect feeding trials were performed as previously described [26]. This assay was conducted with three biological replicates and three technical replicates, and its results were analyzed with the Student’s *t*-test.

## 4. Conclusions

We successfully expressed and purified recombinant SaPIN2b protein in *E. coli*, and our results demonstrated that SaPIN2b could effectively inhibit the activities of serine proteinases, including subtilisin A, chymotrypsin, trypsin, and insect midgut proteinases. In addition, the overexpression of SaPIN2b in transgenic tobacco plants enhanced the resistance of these plants to *H. armigera*. Taken together, our observations suggest that SaPIN2b, a PIN2 proteinase inhibitor that was isolated from a weed, the nightshade *Solanum americanum,* is a potent serine proteinase inhibitor that could be used as a valuable gene to produce insect-resistant transgenic plants.

## Figures and Tables

**Table 1:** The inhibitory effect of rSaPIN2b on the activity of various proteinases.

Proteinase (final concentration)	rSaPIN2b (nM)	I/E a	Inhibition (nM) b	Substrate (final concentration)	IC_50_
Subtilisin A (83 nM)	**74**	**0.9**	**74.3**	Succinylcasein (14 nM)	**7.3**
Chymotrypsin (25 nM)	**41.6**	**1.7**	**54**	BTEE (0.5 mM)	**34.9**
Trypsin (7.5 nM)	**131.2**	**17.5**	**61**	TAME (1 mM)	**126.7**

a *I*/*E* is the ratio of rSaPIN2b concentration to proteinase concentration.

b Inhibition (%) = [1 − (velocity in the presence of inhibitor/velocity of uninhibited control)] × 100%.

**Table 2:** The mortality and pupation rate of *Helicoverpa armigera* larvae that fed on control and SaPIN2b-overexpressing tobacco plants. Ten early second-instar *H. armigera* larvae were introduced to transgenic and wild type (WT) plants. VO, vector-only transgenic plants; T1 and T5, SaPIN2b-overexpressing plants. Three biological replicates were performed with three technical replicates for each treatment.

	Mortality (%)	Pupation rate (%)
WT	23.3 ± 5.8	78.6 ± 6.2
VO	30.0 ± 13.3	81.5 ± 3.2
T1	56.7 ± 5.8 ^**^	38.3 ± 12.6 ^**^
T5	50.0 ± 10 ^**^	41.1 ± 8.4 ^**^

The asterisks denote significant differences from the control plants (*t*-test, ^**^ indicates that *p* < 0.01).
